# An LEO Constellation Early Warning System Decision-Making Method Based on Hierarchical Reinforcement Learning

**DOI:** 10.3390/s23042225

**Published:** 2023-02-16

**Authors:** Yu Cheng, Cheng Wei, Shengxin Sun, Bindi You, Yang Zhao

**Affiliations:** School of Aeronautics, Harbin Institute of Technology, Harbin 150006, China

**Keywords:** LEO constellation early warning system, hypersonic vehicles, hierarchical policy, multi-agent proximal policy optimization, intelligent decision-making algorithm

## Abstract

The cooperative positioning problem of hypersonic vehicles regarding LEO constellations is the focus of this research study on space-based early warning systems. A hypersonic vehicle is highly maneuverable, and its trajectory is uncertain. New challenges are posed for the cooperative positioning capability of the constellation. In recent years, breakthroughs in artificial intelligence technology have provided new avenues for collaborative multi-satellite intelligent autonomous decision-making technology. This paper addresses the problem of multi-satellite cooperative geometric positioning for hypersonic glide vehicles (HGVs) by the LEO-constellation-tracking system. To exploit the inherent advantages of hierarchical reinforcement learning in intelligent decision making while satisfying the constraints of cooperative observations, an autonomous intelligent decision-making algorithm for satellites that incorporates a hierarchical proximal policy optimization with random hill climbing (MAPPO-RHC) is designed. On the one hand, hierarchical decision making is used to reduce the solution space; on the other hand, it is used to maximize the global reward and to uniformly distribute satellite resources. The single-satellite local search method improves the capability of the decision-making algorithm to search the solution space based on the decision-making results of the hierarchical proximal policy-optimization algorithm, combining both random hill climbing and heuristic methods. Finally, the MAPPO-RHC algorithm’s coverage and positioning accuracy performance is simulated and analyzed in two different scenarios and compared with four intelligent satellite decision-making algorithms that have been studied in recent years. From the simulation results, the decision-making results of the MAPPO-RHC algorithm can obtain more balanced resource allocations and higher geometric positioning accuracy. Thus, it is concluded that the MAPPO-RHC algorithm provides a feasible solution for the real-time decision-making problem of the LEO constellation early warning system.

## 1. Introduction

The space-based tracking problem of hypersonic glide vehicles (HGVs) has received considerable attention in recent years. Compared with ground-based early warning systems, space-based early warning systems have wider spatial coverage and better tracking coherence, and are not limited by geographic location [1]. Space-based early warning systems can detect and track near-Earth vehicles, such as ballistic missiles and HGVs in near real-time conditions.

This is why the United States proposed the idea of a space-based tracking layer. As an important component of the U.S. national defense space architecture (NDSA) and next-generation overhead persistent infrared system (NG-OPIR), the space-based tracking layer is an important component of early warning, tracking, and surveillance of missile targets, such as HGVs. Space-based early warning satellite tracking of HGVs is a coherent tracking decision-making process for multi-targets using multi-satellites [2].

The LEO-constellation-tracking system is a scheduling system for space-based early warning satellites that track targets. The LEO-constellation-tracking system uses infrared sensors on satellites to cooperatively track and point at moving targets to obtain observation angles. It later uses the dual-satellite positioning technique to estimate the target’s position. Therefore, we investigated the cooperative autonomous intelligent decision-making algorithm for the early warning system of the LEO constellation.

The framework structure of mission planning methods commonly used in LEO-constellation-tracking systems includes three main types: centralized, distributed, and decentralized [3]. The centralized mission planning structure requires using a master controller as the mission’s decision-making center, and it is responsible for scheduling the tasks of multi-satellites [4,5,6]. Zhao et al. proposed an improved bat algorithm to solve large-scale multi-objective combinatorial optimization problems [7].

Jinming et al. proposed a hybrid genetic parallel Tabu algorithm to solve the collaborative scheduling problem of multiple resources [8]. Jiang et al. used a two-population artificial bee colony algorithm and a heuristic mission-scheduling algorithm to implement joint observation satellite mission scheduling [9]. In the centralized structure, the satellites do not have independent decision-making capabilities. They need the information interaction center to collect messages from all satellites and coordinate planning. Communication delay is high, and real-time decision-making capabilities are weak.

In the distributed architecture, the mission initiator is the decision center, and after collecting messages from each satellite, the mission decision-making center assigns tasks to the relevant satellites [10]. The distributed architecture distributes the calculation’s load to the multi-satellite, effectively improving the satellite’s real-time decision-making capability. However, there is still a delay caused by uneven load distributions. In the decentralized structure, there is no information interaction center or mission decision center, and each satellite is equipped with independent decision-making and information-sharing capabilities.

To finally achieve the global goal, each satellite follows an identical logical process, including constraint judgment, self-decision, and inter-satellite consensus. The autonomy of the satellite makes the decentralized structure more robust to the uncertainty factors of the target [11]. A data-driven parallel scheduling method was proposed by Du et al. to design probabilistic models and task-allocation methods for the parallel scheduling of multi-satellites in a decentralized structure with a single satellite [12]. Zhou et al. proposed a quick distributed multi-model fusion tracking framework to achieve the cooperative and continuous multi-satellite tracking of HGVs [13].

The local consensus algorithm uses a local-to-global decision process, considering that satellites do not have access to real-time global information at every moment. Satellites can immediately collect global information before making decisions. In the intelligent decision-making algorithm, satellites use artificial intelligence techniques to learn and make decisions autonomously. The intelligent decision-making algorithm has better adaptability for complex and variable tasks. In this paper, we use a decentralized structure to design a reinforcement-learning-based intelligent decision-making method based on the information-sharing and decision-making processes of the consensus algorithm.

Machine learning has shown some advantages in satellite mission planning and scheduling problems [14,15,16]. Deep reinforcement learning is an effective method for solving the sequential decision problem. It can effectively solve difficult sample data acquisition problems in the constellation’s early warning system by continuously updating its decision network via the interaction between the intelligence and the environment. Commonly used deep-reinforcement-learning algorithms currently include the following: deep Q-network (DQN) [17,18,19,20], deep deterministic policy gradient (DDPG) [21,22,23], proximal policy optimization (PPO) [24,25,26], and soft actor–critic (SAC). These algorithms are widely used in the cooperative positioning of moving targets, agile Earth observation satellite mission scheduling, and the collaborative scheduling of ground satellites.

Wei et al. implemented the collaborative scheduling of agile remote sensing satellites using a neural network with an encoder–decoder architecture and an actor–critic (AC) reinforcement-learning framework [27]. Huang et al. used the DDPG algorithm to solve a time-continuous satellite task-scheduling problem [28]. The PPO algorithm is a model-free deep-reinforcement-learning algorithm [29]. This algorithm has low arithmetic power requirements and fast convergence and is the main algorithm used by OpenAI for deep reinforcement learning.

Li et al. effectively solved the satellite resource and time-window-conflict problems in constellation task scheduling using the PPO algorithm [26]. The decision-making process of large-scale constellations is collaborative and coherent, and the state information of satellites, sensors, and targets changes in real time; therefore, the solution space of the decision-making algorithm is highly dimensional. Reducing the solution space of the decision-making algorithm is an effective way to improve the real-time performance of the decision-making algorithm.

Hierarchical reinforcement learning uses the decomposition of objectives into sub-objectives, which can effectively reduce the solution space and is an effective method for solving large-scale constellation decision-making problems [30]. Ren et al. proposed a hierarchical reinforcement-learning algorithm based on Q-learning for response speed and stability problems in randomly occurring urgent tasks [31]. Zhao et al. proposed a two-stage neural network combinatorial optimization method based on DDPG to solve the problem of temporal task assignments in dynamic environments [32].

Yue et al. proposed a hierarchical multi-agent reinforcement learning (HMARL) that solves the target assignment problem using a multi-agent deep Q-network (MADQN). The task assignment problem in the execution phase is then solved using independent asynchronous proximal policy optimization (IAPPO) [33]. Guo et al. proposed a multi-agent reinforcement-learning algorithm based on PPO by introducing a centralized training and decentralized execution framework, which can obtain a decentralized policy for each satellite [34]. Reinforcement learning has excellent performance in terms of calculation efficiency and adaptability to dynamic environments.

The local search algorithm showed excellent performance in optimizing the sensor over the process in coherent observations [35]. Adding local search algorithms to reinforcement learning can further enhance the search capability of the algorithm based on the above advantages. He et al. developed a two-stage scheduling algorithm framework that combines Q-learning and traditional mixed-integer programming to achieve multi-satellite task scheduling [18].

In this paper, we focus on the intelligent decision-making algorithm for HGVs in the LEO constellation’s early warning system, and the main contributions are as follows:

(1) The constellation configuration of the constellation warning system is introduced, and the configuration parameters are selected with the objective of proximity space coverage. The constraints of sensor and communication are considered, and the visible range of the sensor is calculated according to the target infrared radiation intensity, the proximity observation, and other factors. The multi-target gaze-tracking model and dual-satellite geometric positioning model of the constellation early warning system are established.

(2) A hierarchical proximal policy-optimization algorithm is designed to address the problem of large solution spaces for large-scale constellation mission decision making, where the upper layer network focuses on selecting sensor search regions and the lower layer network focuses on selecting tracking targets. The hierarchical decision-making approach effectively reduces the solution space, improves the decision-making efficiency, and maximizes the global gain while uniformly distributing the satellite resources.

(3) A single-satellite local search method based on random hill climbing is added after the hierarchical proximal policy optimization to improve the optimal search capability of the intelligent decision-making algorithm. The satellite search in the visible targets can improve the positioning accuracy of the dual-satellite combination. The random-hill-climbing algorithm takes the decision results of the hierarchical proximal optimization strategy as the premise, which improves the search capability of the intelligent decision algorithm while ensuring that the intelligent decision algorithm still has the performance of near real-time decision making.

The following sections of this paper are as follows: Section 2 describes the modeling of the gaze-tracking problem of HGVs in LEO constellation warning systems, including constraints, such as inter-satellite communication and infrared sensors, dual-satellite geometric positioning algorithms, geometric dilution of precision (GDOP), and constellation information-sharing strategies. Section 3 presents the state processing method and network design of the hierarchical proximal policy-optimization algorithm, incorporating random hill climbing (MAPPO-RHC).

A detailed description of the regional decision-making network, the target decision-making network, and the single-satellite local search method based on random hill climbing is provided. Section 4 details two scenarios in the simulation system to analyze and evaluate the tracking and positioning results of the constellation for coherently generated HGVs, and it verifies the capability of MAPPO-RHC regarding the coverage numbers and positioning accuracy. Section 5 summarizes the content, limitations, and future extension directions of the research in this paper.

## 2. Problem Description

In the gaze-tracking problem of HGVs for the constellation’s early warning system, the satellites cannot accomplish the full-time tracking task of the target independently, so multi-satellites need to collaborate in order to coherently track a target [36]. Both satellites and targets are in high-speed motion, and thus the spatial relationship between satellites and targets dynamically changes [37].

Since HGVs are non-cooperative, it is difficult for satellites to accurately predict their future trajectories. Multiple multi-satellites observing the same target simultaneously from different angles can avoid the target loss problem due to target maneuvers. Thus, it can be observed that the cooperative positioning process in the near space of the LEO constellation’s early warning system is a comprehensive process that includes multi-satellite cooperation, multi-coverage, and high time-effective decision-sharing characteristics.

### 2.1. LEO Constellation Early Warning System

This section establishes the constellation model using three aspects: constellation configuration, inter-satellite communication constraints, and infrared sensor constraints. Then, the coherent gaze-tracking problem of HGVs by LEO constellation warning systems is described, including the description of GDOP and dual-satellite positioning methods. Finally, the information-sharing policy of the constellation is described.

#### 2.1.1. Constellation Configuration

The LEO constellation early warning system researched in this paper uses hypersonic vehicles as observation targets. It requires global full-time coverage; thus, the Walker-δ constellation’s configuration is suitable. The constellation configuration consists of multi-satellites with the same orbital inclination and orbital altitude. The phases of the satellites in each orbital plane of the constellation are uniformly distributed, and the ascending nodes between the orbital planes are uniformly distributed. The Walker-δ constellation’s configuration can be expressed as N/P/F, where *N* is the number of satellites, *P* is the number of orbital planes, and *F* is the phase difference between adjacent orbital planes.

Near space is the airspace between 20 and 100 km from the ground. The onboard infrared (IR) sensor is constrained by limb observations; it has a short visible time window for near-space vehicles. The position of a target can be determined when more than two IR sensors cover it simultaneously. Therefore, the LEO constellation warning system needs to meet the number of HGVs that can achieve more than twice the coverage for different regions of the world. In this paper, a constellation configuration of 152/8/4 is used. The orbital inclination of the satellite is 70 degrees. This constellation configuration can meet the coverage requirement of HGVs.

#### 2.1.2. Inter-Satellite Communication Constraints

While the LEO constellation warning system can cooperate with the transport layer satellites to realize information communication with lower delay, the LEO constellation warning system also needs to have inter-satellite communication capabilities. Therefore, considering the real-time connectivity requirement of the constellation communication network, the constraints on the inter-satellite links are described as follows.

**Figure 1:**
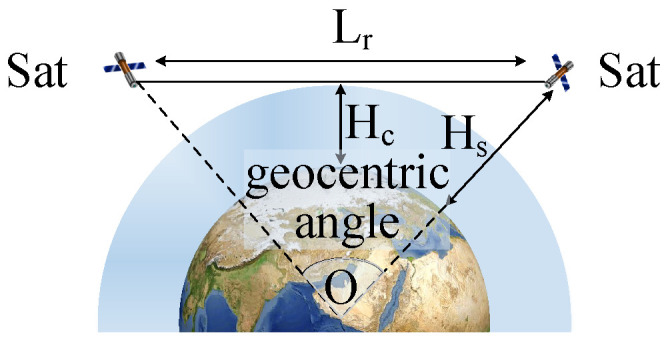
Inter-satellite communication.

As shown in Figure 1, two adjacent satellites use laser communication technology. In order to reduce link losses, the communication link needs to avoid crossing the atmosphere. Considering the minimum link height, $H_{c}$, the maximum inter-satellite communication distance can be expressed as follows:

(1) $$L_{r}=2\cdot \left(H_{s}+R_{e}\right)\cdot \sin\left(\frac{\alpha_{\max}^{isl}}{2}\right)$$

where $H_{s}$ is the orbital altitude of the satellite, $R_{e}$ is the Earth’s radius length, and $\alpha_{\max}^{isl}$ is the maximum geocentric angle.

(2) $$\alpha_{\max}^{isl}=2arcos\left(\frac{H_{c}+R_{e}}{H_{s}+R_{e}}\right)$$

#### 2.1.3. Infrared Sensor Constraint

Each satellite in the LEO constellation early warning system carries a condensing mid-wave IR sensor that captures the infrared radiation generated by the friction between HGVs and the atmosphere. Gaze-based infrared sensors have a narrow field of view and flexible pointing. This paper assumes that one sensor can only track one target simultaneously. The sensor needs to satisfy geometric visibility, limb observation, and maximum detection distance constraints due to the influence of infrared radiation from the earth and atmosphere.

**Figure 2:**
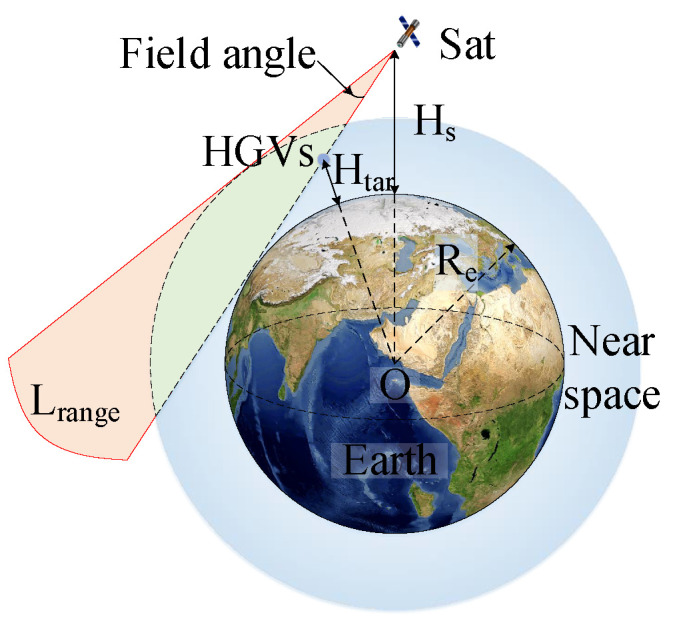
Detection range of infrared sensors.

As shown in Figure 2, the airspace covered by the IR sensor is determined by the field-of-view angle $\alpha_{field}$, the maximum detection distance $L_{range}^{\max}$, the satellite orbit altitude $H_{s}$, and the target flight altitude $H_{tar}$. The geometric visibility constraint specifies that the satellite cannot be obscured by the Earth or other obstacles in its line of sight to the target. The limb observation constraint specifies that the satellite needs to maintain a deep-space background in its observational field of view, with no infrared-emitting objects, such as the Earth or the Sun in the field of view. $L_{range}^{\max}$ is related to the capability of the sensor and the intensity of the target’s infrared radiation, and it is described as follows [38]:

(3) $$L_{range}^{\max}=\frac{{\left[\pi \delta D_{0}^{2}D^{*}I\tau_{a}\tau_{o}\right]}^{1/2\;}}{2SNR_{\min}^{1/2\;}{\left(A_{d}\Delta f\right)}^{1/4\;}}$$

where $D_{0}$ is the aperture diameter of the sensor, $D^{*}$ is the detection of the sensor ($m\cdot Hz^{1/2\;}\cdot W^{-1}$), $\tau_{a}$ is the ambient transmittance, $\tau_{o}$ is the transmittance of the optical system, δ is the sensor signal process factor, $A_{d}$ is the detection unit area (m^2^), Δf is the noise equivalent bandwidth (Hz), ${SNR}_{\min}$ is the minimum signal-to-noise ratio required for the sensor to detect the target, and *I* is the infrared intensity of the target captured by the sensor. The HGVs researched in this paper enter the near space in an unpowered gliding manner, and the friction between the skin and the air generates infrared radiation [39]. Infrared radiation intensity can be expressed as follows [40]:

(4) $$I=\frac{\varepsilon A\cos\theta }{\pi }\int_{\lambda_{1}}^{\lambda_{2}}\frac{C_{1}}{\lambda^{5}\left(e^{C_{2}/\lambda T\;}-1\right)}d\lambda$$

where *A* is the infrared radiated area of the target (m^2^); $\lambda_{1}$ and $\lambda_{2}$ are the lower and upper limits of the infrared band, respectively; ε is the spectral emissivity of the target surface; $c_{1}$ is the first radiation constant (W·m^2^); and $c_{2}$ is the second radiation constant (m·K). The stagnation temperature, *T*, is described as follows:

(5) $$T_{s}=T_{0}\left[1+\beta \left(\frac{\nu -1}{2}\right)M^{2}\right]$$

where $T_{0}$ is the ambient temperature of the target position, ν is the atmospheric adiabatic index, β is the recovery coefficient of heat transfer, and *M* is the Mach number of the target.

In addition, the sensor has different maneuvering capabilities in standby, search, and imaging states. In this paper, to simplify the problem, the sensor is assumed to switch between two states: search and imaging. The maximum rotational speed of the sensor in the search state is defined as $\omega_{search}^{\max}$; the maximum rotational speed of the sensor in the imaging state is $\omega_{sta}^{\max}$ in order to ensure infrared imaging accuracies.

### 2.2. LEO Constellation Early Warning System Near-Space Multi-Target Tracking Problem

**Figure 3:**
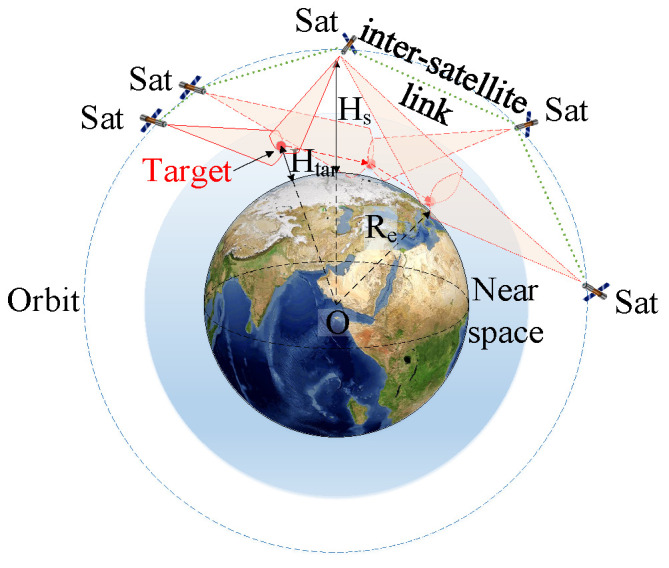
The LEO constellation warning system collaborative tracking.

In the tracking process of HGVs, the LEO constellation early warning system coherently covers the target via the cooperative observation of multi-satellites. Since the LEO satellites and HGVs are in high-speed motion, the position relationship between satellites and targets is highly dynamic. As shown in Figure 3, multi-satellites must track the target coherently during flight to achieve multi-coverage. For the LEO constellation warning system, HGVs are non-cooperative targets, and their positions and velocities are obtained by constellation observations and message sharing.

### 2.3. Dual Satellite Geometric Positioning

The LEO constellation’s early warning system needs at least two satellites to track the same target simultaneously in order to obtain the target’s position. Therefore, the multi-satellite, multi-angle tracking of observation targets is a central task in the decision-making process of the constellation’s warning system.

Assume that the two satellite positions for co-observation are $\left[\begin{array}{ccc} X_{a} & Y_{a} & Z_{a} \end{array}\right]$ and $\left[\begin{array}{ccc} X_{c} & Y_{c} & Z_{c} \end{array}\right]$. The farthest detectable points in the satellite’s line of sight, $\left[\begin{array}{ccc} X_{b} & Y_{b} & Z_{b} \end{array}\right]$ and $\left[\begin{array}{ccc} X_{d} & Y_{d} & Z_{d} \end{array}\right]$, are calculated from the satellite’ss positions and observation angles. The observation directions of two satellites due to observation angle errors are usually anisotropic straight lines. The common perpendicular of two straight lines in different planes is calculated first, and then the common plumb line is divided proportionally to estimate the target’s position. The positioning algorithm is as follows:

(6) $$\left\{\begin{array}{c} F_{1ab}={\left(X_{b}-X_{a}\right)}^{2}+{\left(Y_{b}-Y_{a}\right)}^{2}+{\left(Z_{b}-Z_{a}\right)}^{2}\\ F_{1cd}={\left(X_{d}-X_{c}\right)}^{2}+{\left(Y_{d}-Y_{c}\right)}^{2}+{\left(Z_{d}-Z_{c}\right)}^{2}\\ F_{2}=\left(X_{b}-X_{a}\right)\left(X_{d}-X_{c}\right)+\left(Y_{b}-Y_{a}\right)\left(Y_{d}-Y_{c}\right)+\left(Z_{b}-Z_{a}\right)\left(Z_{d}-Z_{c}\right)\\ F_{3ab}=\left(X_{b}-X_{a}\right)\left(X_{c}-X_{a}\right)+\left(Y_{b}-Y_{a}\right)\left(Y_{c}-Y_{a}\right)+\left(Z_{b}-Z_{a}\right)\left(Z_{c}-Z_{a}\right)\\ F_{3cd}=\left(X_{d}-X_{c}\right)\left(X_{c}-X_{a}\right)+\left(Y_{d}-Y_{c}\right)\left(Y_{c}-Y_{a}\right)+\left(Z_{d}-Z_{c}\right)\left(Z_{c}-Z_{a}\right) \end{array}\right.$$

(7) $$\left\{\begin{array}{c} t_{1}=\frac{F_{3ab}\cdot F_{1cd}-F_{3cd}\cdot F_{2}}{F_{1ab}\cdot F_{1cd}-F_{2}^{2}}\\ t_{2}=\frac{F_{3cd}\cdot F_{1ab}-F_{3cd}\cdot F_{2}}{F_{2}^{2}-F_{1ab}\cdot F_{1cd}}\\ X_{tar}=0.5\left\{\left[X_{a}+t_{1}\left(X_{b}-X_{a}\right)\right]+\left[X_{c}+t_{2}\left(X_{d}-X_{c}\right)\right]\right\}\\ Y_{tar}=0.5\left\{\left[Y_{a}+t_{1}\left(Y_{b}-Y_{a}\right)\right]+\left[Y_{c}+t_{2}\left(Y_{d}-Y_{c}\right)\right]\right\}\\ Z_{tar}=0.5\left\{\left[Z_{a}+t_{1}\left(Z_{b}-Z_{a}\right)\right]+\left[Z_{c}+t_{2}\left(Z_{d}-Z_{c}\right)\right]\right\} \end{array}\right.$$

where *F* and *t* are intermediate variables, and $\left[\begin{array}{ccc} X_{tar} & Y_{tar} & Z_{tar} \end{array}\right]$ is the estimated target position.

**Figure 4:**
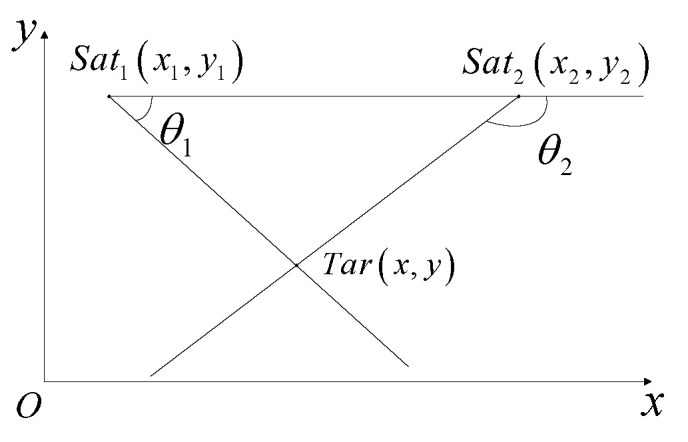
Dual-satellite positioning.

The image plane measurement error and Euler angle measurement error of the onboard IR sensor can be equated to the error of the observation angle in the two-dimensional plane. By projecting the satellite’s line of sight into the plane that is normal relative to the common perpendicular, the observation angle positioning method for the two-dimensional plane is shown in Figure 4.

The observation angle is expressed as follows.

(8) $$\left\{\begin{array}{c} \theta_{1}=\arctan\left(\frac{y-y_{1}}{x-x_{1}}\right)\\ \theta_{2}=\arctan\left(\frac{y-y_{2}}{x-x_{2}}\right) \end{array}\right.$$

The coordinates of the projection of the target in the plane are $\left[x,y\right]$. $\left[x_{1},y_{1}\right]$ and $\left[x_{2},y_{2}\right]$ are the projection coordinates of the two satellites in the plane, respectively. From the geometric relationship in Figure 4, it follows that

(9) $$\left\{\begin{array}{c} x=\frac{y_{2}-y_{1}+x_{1}\tan\theta_{1}-x_{2}\tan\theta_{2}}{\tan\theta_{1}-\tan\theta_{2}}\\ y=\frac{y_{2}\tan\theta_{1}-y_{1}\tan\theta_{2}+\left(x_{1}-x_{2}\right)\tan\theta_{1}\tan\theta_{2}}{\tan\theta_{1}-\tan\theta_{2}} \end{array}\right.$$

The observation matrix, $H_{\theta }$, for the conversion from the observed angle error to the target’s positioning error is as follows:

(10) $$H_{\theta }=\left[\begin{array}{cc} \frac{\partial x}{\partial \theta_{1}} & \frac{\partial x}{\partial \theta_{2}}\\ \frac{\partial y}{\partial \theta_{1}} & \frac{\partial y}{\partial \theta_{1}} \end{array}\right]=\frac{L}{2\sin^{2}\left(\theta_{2}-\theta_{1}\right)}\left[\begin{array}{cc} \sin\left(2\theta_{2}\right) & -\sin\left(2\theta_{1}\right)\\ 2\sin^{2}\left(\theta_{2}\right) & -2\sin^{2}\left(\theta_{1}\right) \end{array}\right]$$

where *L* is the distance between the two satellites.

The GDOP is calculated as follows:

(11) $$GDOP_{\theta }=\sqrt{Trace\left(H_{\theta }\left[\begin{array}{cc} \sigma_{\theta }^{2} & 0\\ 0 & \sigma_{\theta }^{2} \end{array}\right]H_{\theta }^{T}\right)}=\frac{L\sigma_{\theta }\sqrt{\sin^{2}\theta_{1}+\sin^{2}\theta_{2}}}{\sin^{2}\left(\theta_{2}-\theta_{1}\right)}$$

where $\sigma_{\theta }$ is the error of the observation angle.

### 2.4. Constellation Information Sharing Policy

The constellation adopts a decentralized information-sharing architecture. Each satellite has independent decision-making and information-sharing capabilities. The satellites are interconnected through an inter-satellite communication network. Each satellite has six information vectors, including the mission bundle, plan sequence, execution time sequence, winner list, bid list, and timestamp vector. The task bundle includes the set of tasks that the satellite has selected and decided successfully among known targets, and the tasks are listed in the order of successful decisions.

The targets in the plan sequence are the same as in the task bundle, and the targets are listed in the order of execution. The execution time sequence includes tracking the start time of each target in the decision-making result. The winner list includes the numbers of bid-winning satellites for known targets. The bid list includes the actual bid information of the winner. The timestamp vector includes the schedule for receiving information about adjacent satellites and updating local information.

**Figure 5:**
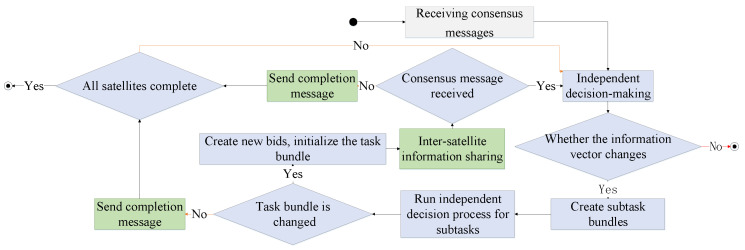
Inter-satellite information-sharing policy.

The constellation information-sharing policy consists of two iterative phases: the independent decision-making phase and the consensus phase. Satellites independently select tracking targets and calculate evaluation values for known target information and locally stored information vectors in the independent decision-making phase. Then, satellites share the bid information with adjacent satellites and receive shared information through an inter-satellite communication network in the consensus phase. These two phases are iterated until convergence. The flow of inter-satellite information-sharing policies is shown in Figure 5:

The iterative loop of independent decision-making and consensus processes in the constellation’s information-sharing policy process enables real-time decision making for dynamic tasks. The speed of decision making and the reward are influenced by the decision-making algorithm. Early warning constellations require near real-time decision-making methods that can be considered for multi-satellite collaboration. In the next section, decision-making algorithms based on hierarchical proximal policy optimization are built to solve the independent decision-making problem of satellites in the LEO constellation’s early warning systems.

## 3. Decision-Making Approach Based on Hierarchical Proximal Policy Optimization

The cooperative positioning process of HGVs by the LEO constellation’s early warning system is a dynamic task scheduling process involving multi-satellite and multi-targets. The task assignment of early warning satellites is correlated with time series and dynamic, and therefore the decision-making algorithm faces the problem of large solution space and falls into the local optimum. The multi-agent proximal policy-optimization algorithm (MAPPO) is used as an improved base algorithm.

The MAPPO algorithm employs a centralized training distributed execution AI framework suitable for this fully cooperative relationship in multi-satellite collaborative decision making scenarios where satellites cooperate fully with each other. Multiple satellites jointly perceive and independently make decisions. MAPPO uses global data to train satellites and share reward functions among satellites, which can enhance the multi-satellite collaborative capability. Moreover, MAPPO adopts on-policy update strategy, which has higher training efficiency.

In this section, a hierarchical proximal policy-optimization algorithm is designed using a state-space-partitioning method to reduce the solution space. The external reward is maximized, and the pointing sequence of the satellite is optimized when tracking the target. Finally, a local search algorithm based on random hill climbing is introduced to effectively improve the search capability of the decision-making algorithm in the solution space.

The MAPPO-RHC algorithm designed in this section is a hierarchical reinforcement learning based constellation independent decision-making algorithm. The algorithm implements multi-to-multi task assignments in a dynamic, multi-constrained, and multi-objective task environment. Combining hierarchical reinforcement learning with local search algorithms exploits the inherent advantages of reinforcement learning in real time and improves the search capability of the algorithm.

### 3.1. Multi-Agent Proximal Policy Optimization Algorithm

Due to the characteristics of distributed decision-making for homogeneous satellites, this paper adopts the multi-agent proximal policy-optimization algorithm (MAPPO) as the decision-making algorithm for early warning satellites. Each satellite has a set of actor-critic networks. In the centralized training structure, experience is shared among satellites. Independent strategies are trained for each satellite’s actor network using global information. The goal of the policy is to optimize the sensor pointing sequence to maximize the coherent tracking of the target trajectory and to optimize the observation angle’s distribution to improve the geometric positioning accuracies during multi-satellite cooperative positioning.

The LEO constellation cooperative decision-making problem is defined as $\Gamma =\langle S$, A, P, r, N, $\gamma \rangle$. $s_{t}\in S$ is the environment state, and *t* is the time step. Each satellite $u\in N=\left\{1,2,\ldots ,N\right\}$ chooses an action $a_{t}\in A\equiv A^{N}$ in each time step. P is the state transfer function. The same reward function, $r\left(s_{t},a_{t}\right)$, is used for all satellites. $\gamma \in \left[0,1\right)$ is the discount factor.

#### 3.1.1. Environmental State Space

The environmental state space, *S*, is defined as $S=\{T_{num}$, ${Obs}_{angle}$, ${Pos}_{tar}$, ${Vel}_{tar}$, $L_{tar}$, $L_{score}\}$, $T_{num}$ is the number of targets, $Obs_{sat_{n}}=[\alpha_{}^{azi}$, $\alpha_{}^{pit}]$ is the observation angle, and *n* is the satellite number. ${Pos}_{tar}$ is the position of the target, and ${Vel}_{tar}$ is the velocity of the target. $L_{tar}=\left\{{Tar}_{{sat}_{1}}\right.$, ${Tar}_{{sat}_{2}}$, *…*, $\left.{Tar}_{{sat}_{n}}\right\}$ is the list of targets tracked by satellites, and ${Tar}_{{sat}_{n}}$ is the number of the target pointed by the sensor of satellite ${sat}_{n}$. $L_{score}=\left\{C_{{sat}_{1}}^{{ID}_{tar}}\right.$, $C_{{sat}_{2}}^{{ID}_{tar}}$, *…*, $\left.C_{{sat}_{n}}^{{ID}_{tar}}\right\}$ is the list of satellite rewards, and $C_{{sat}_{n}}^{{ID}_{tar}}$ is the reward obtained when the satellite’s (${sat}_{n}$) sensor points to target ${ID}_{tar}$. The dimensions of $L_{tar}$ and $L_{score}$ are the same as the number of satellites, and ${Tar}_{{sat}_{n}}=0$ and $C_{{sat}_{n}}^{{ID}_{tar}}=0$ when the target is not observed by satellite ${sat}_{n}$.

#### 3.1.2. Action Space

The action space, $A=\left\{Tar_{1}\right.$, $Tar_{2}$, *…*, $\left.Tar_{k}\right\}$, of the satellite is described as the target pointed by the sensor at *t*. A is a *k*-dimensional vector, and the initial value of $Tar_{k}$ is −1. Since the action space contains an action pointing to the initial position in addition to the target direction, A contains the direction of all visible targets when the number of visible targets of the satellite is less than k−1. Conversely, A contains the directions of the top k−1 targets among the visible targets in descending order of the reward. The satellite considers the current pointing of the sensor and the environment state together to decide where the sensor points toward at the next moment.

#### 3.1.3. Reward Function

The reward function is used to evaluate the value of the satellite’s actions. $R_{t}$ is obtained by $a_{t}$, and $s_{t}$ and is calculated as follows:

(12) $$r_{t}=I_{vis}\left(C_{m}\cdot \left|L_{range}^{\max}-L_{range}^{tar}\right|+C_{a}\cdot \frac{\omega^{\max}}{\alpha_{cur}^{tar}+\omega^{\max}}\right)$$

where $C_{m}$ and $C_{a}$ are the weights of observation angle and sensor rotation, respectively. $L_{range}^{max}$ is the maximum detection distance of the sensor, and $L_{range}^{tar}$ is the distance between the satellite and the target. $\alpha_{cur}^{tar}$ is the rotation angle of the sensor from the current direction to the target direction, and $\omega^{\max}$ is the maximum angular velocity of the sensor rotation. The visibility parameter $I_{vis}$ is expressed as follows.

(13) $$I_{vis}=\left\{\begin{array}{c} 1,Visibility=True\\ 0.2,Visibility=False \end{array}\right.$$

When the sensor is visible to the target, the value of $I_{vis}$ is 1. Conversely, in order to give the free sensor the capability to point near the target in advance, $I_{vis}$ is set to 0.2.

#### 3.1.4. Network Update

The decision-making network gradient update equation is as follows:

(14) $$\hat{g}={\hat{E}}_{t}\left[\nabla_{\theta }\log\pi_{\theta }\left(a_{t}|s_{t}\right){\hat{A}}_{t}\right]$$

where policy $\pi_{\theta }\left(a_{t}|s_{t}\right)$ denotes the probability of the choice of action $a_{t}$ by the agent after obtaining environmental state $s_{t}$ at time step *t*. $\hat{E}$ is the average of the samples at time step *t*. ${\hat{A}}_{t}$ is the generalized advantage function at time step *t*:

(15) $${\hat{A}}_{t}=\sum_{l=0}^{\infty }{\left(\gamma \lambda \right)}^{l}\left(r_{t}+\gamma V\left(s_{t+l+1}\right)-V\left(s_{t+l}\right)\right)$$

where γ is the discount factor, and λ is the discount factor of generalized advantage estimation (GAE). $r_{t}$ is the current reward, and V is the critic network’s evaluation value.

The objective function of the policy gradient is as follows:

(16) $$L\left(\theta \right)={\hat{E}}_{t}\left[\min\left(\frac{\pi_{\theta }\left(a_{t}|s_{t}\right)}{\pi_{\theta old}\left(a_{t}|s_{t}\right)}\cdot {\hat{A}}_{t},clip\left(\frac{\pi_{\theta }\left(a_{t}|s_{t}\right)}{\pi_{\theta old}\left(a_{t}|s_{t}\right)}\left(\theta ,1-\varepsilon ,1+\varepsilon \right)\cdot {\hat{A}}_{t}\right)\right)\right]$$

where $\frac{\pi_{\theta }\left(a_{t}|s_{t}\right)}{\pi_{\theta old}\left(a_{t}|s_{t}\right)}$ is the importance weight. The role of the importance weight is to adjust the reward by using the ratio of the old and new policy gradients. Actions that are more likely to be taken are given higher weights, and actions that are less likely to be taken are given lower weights. The importance weight is restricted by hyper-parameter ε and the truncation operation, CLIP, applied to $\left(1-\varepsilon ,1+\varepsilon \right)$.

The updated equation for the policy gradient is as follows:

(17) $$\theta_{new}=\theta_{old}+\alpha \hat{g}$$

where α is the parameter update step.

**Figure 6:**
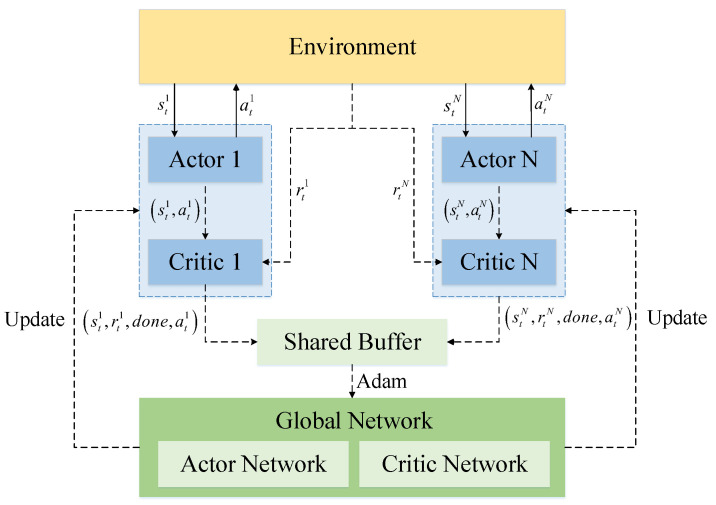
MAPPO’s parameter update.

The MAPPO algorithm’s training and parameter updating use an experience-sharing mechanism. Global information is used to train independent decision-making policies for each satellite. The parameter update process is shown in Figure 6.

The algorithm includes *N* local policy networks and a global policy network. In each time step *t*, the algorithm calculates action policy $a_{t}^{N}$ for *N* actor networks and obtains the reward, $r_{t}^{N}$, of the action after interacting with the environment. Each local policy network calculates $T_{e}$ steps. Then, the advantages, $\hat{A}{}_{t}$, t=1, 2, 3, *…*, $N\cdot T_{e}$, are calculated. *N* local networks share the experience $\left(s_{t}^{N}\right.$, $r_{t}^{N}$, done, $\left.a_{t}^{N}\right)$ and then update the global network using the Adam optimizer. Finally, local networks copy the new global network to each local network.

The MAPPO-RHC algorithm researched in this paper also uses the same experience-sharing mechanism as MAPPO. The satellites researched in this paper are isomorphic, each with the same orbital altitude and infrared sensor. Therefore, this mechanism is scalable and can adapt to the increase or decrease in the number of satellites. The parameters of the staged network during the training process can be used as the base network for the newly added satellites, and the parameters continue to be trained.

### 3.2. MAPPO-RHC Algorithm

#### 3.2.1. Hierarchical Decision-Making Network Design

There are problems with large solution spaces and complex decision-making in the collaborative tracking process of the LEO constellation. Moreover, satellites searching for a large solution space based on shared information in a distributed structure are prone to fall into a local optimum. As of the above reasons, hierarchical decision-making networks are established. The two-layer network maximizes macroscopic and microscopic rewards and reduces the solution space for satellite decision making. The decision-making network is divided into a region and a target decision-making network using the hierarchical approach to partition the state space, $S_{i}$.

The region’s decision-making network is the upper-layer network that divides the search direction of the satellite into $N_{region}$ regions that do not overlap each other. Based on the distribution of target positions and the utilization of satellite resources to judge the macroscopic situation, the sensor’s search region is selected. The target decision-making network is the lower-layer network, which is driven by the upper-layer network.

The lower-layer network determines the search region based on the search direction provided by the upper-layer network. Considering factors, such as observation distance, rotation angle, and the dispersion of the angle at which the target is observed, the lower-layer network selects the target within the search region.

The upper-layer network focuses on finding the search region with the greatest macroscopic reward. This network collects targeted situational information and decision-making information from the environment and then outputs the results to the lower-layer network. The upper-layer network does not directly change the external environment and does not have access to feedback from the external environment. Therefore, a policy-processing layer is added between the two decision networks.

The role of the policy-processing layer is to receive the policy output from the upper-layer network and assign internal returns to the lower-layer network. The lower-layer network selects the target based on the state of the environment and the internal reward. The policy-processing layer receives the state of the environment and the selected target from the lower-layer network, calculates the evaluation value of this decision-making step, and feeds it back to the upper-layer network. The upper-layer network iteratively optimizes the action evaluation value based on the feedback.

**Figure 7:**
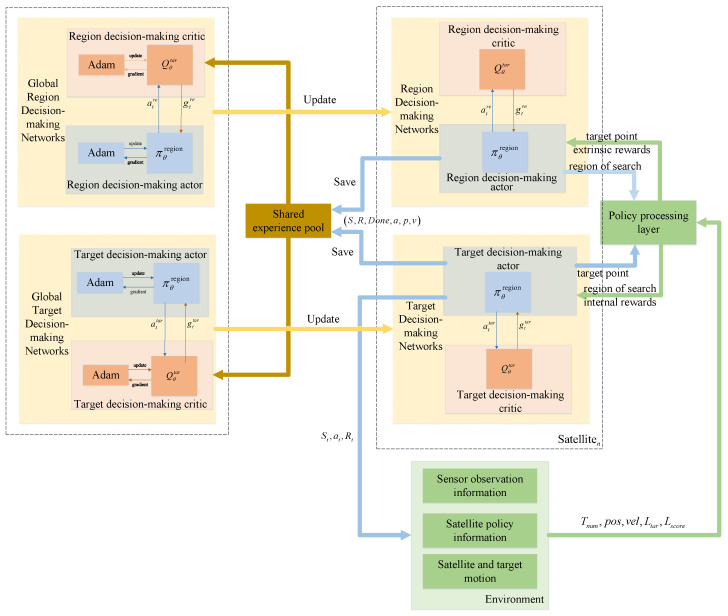
The structure of HPPO.

The hierarchical decision network architecture for each satellite is shown in Figure 7. Each satellite has a local actor network and a local critic network. The experience data from the local network interacting with the environment is stored in a shared experience pool. Then, the global network’s parameters are updated using the Adam optimizer and updated relative to the local network at a fixed period. The flow of the algorithm is as follows.

(1) The upper-layer network obtains the environment state. The satellite obtains information on the target’s observation angle, $S_{t}^{obs}=\left\{{Obs}_{angle}\right\}$, via the sensor at *t*. Then, shared information $S_{t}^{share}=\left\{T_{num}\right.$, $L_{tar}$, $L_{score}$, $\left.L_{obs}\right\}$ is obtained in the inter-satellite communication network.

(2) The action space of the upper-layer network is defined. The dimension of the action space $a_{reg}$ of the upper-layer network is as follows:

(18) $$Dim_{reg}=\frac{360}{R_{unit}}+1$$

where $R_{unit}$ is the region cell.

The upper-layer network splits the search region uniformly in $R_{unit}$, where $R_{unit}$ is the angle that can be divided by 360.

(3) After receiving $a_{reg}$, the policy-processing layer calculates internal reward $R_{\mathrm{int}}$:

(19) $$R_{\mathrm{int}}=\left\{\begin{array}{c} C_{m}\cdot \frac{\sum_{i=1}^{i=n_{c}}c_{o}r_{m}}{n_{in}*r_{m}^{\max}}+C_{r}\cdot \left(R_{\max}^{sen}-R_{\min}^{\mathrm{mi}s}\right),0\leq a_{reg}<6\\ 0,a_{reg}=6 \end{array}\right.$$

where $C_{m}$ and $C_{r}$ are the weight of the angle’s reward and the weight of the distance reward, respectively. $n_{c}$ is the number of visible targets in the $a_{reg}$ region, and $n_{in}$ is the total number of targets in the $a_{reg}$ region. $R_{\max}^{sen}$ is the maximum distance detectable by the sensor, and $R_{\min}^{\mathrm{mis}}$ is the minimum distance from the satellite among the $n_{c}$ targets. $c_{o}$ is the coverage discount factor, $r_{m}$ is the observation angle score, $r_{m}^{\max}$ is the maximum score, and the score table is as follows:

In Table 1, the incident azimuth $\alpha_{az}^{inc}$ represents the projection in the horizontal plane of the angle between the direction from the target to the satellite and the direction of the target’s velocity. Visibility $I_{vis}$ indicates the target visibility from the satellite. $R_{\mathrm{int}}$ calculated by the policy-processing layer is saved to the experience pool along with the training data as the regional decision network’s critic.

(4) The policy-processing layer feeds $a_{reg}$ and $R_{\mathrm{int}}$ into the lower-layer network. The lower-layer network reduces the complete environment state $S_{t}$ to $s_{t}^{tar}$ according to $a_{reg}$. The input of the lower-layer network consists of $s_{t}^{tar}$ and $R_{\mathrm{int}}$, and the dimension of output $a_{tar}$, $N_{max}^{rec}$, is determined by the recognition capability of the sensor. The satellite adjusts the pointing of the sensor according to $a_{tar}$. It saves $a_{tar}$ and latest state $S_{t+1}$ after interacting with the environment in the experience pool, which is then fed back to the policy-processing layer. The external reward, $R_{\mathrm{ext}}$, is obtained by $a_{tar}$, $L_{bit}$, and $L_{win}$ calculations:

(20) $$R_{ext}=C_{m}\cdot \frac{\sum_{i=1}^{i=n_{c}}c_{t}r_{m}}{n_{in}*r_{m}^{\max}}+C_{a}\cdot \frac{1}{\left|\alpha_{Azi}^{cur}-\alpha_{Azi}^{tar}\right|+1}$$

where $c_{t}$ is the discount factor of the incident azimuth distribution, which reduces the rewards for satellites with a similar $\alpha_{az}^{inc}$ and increases the rewards for satellites with larger $\alpha_{az}^{inc}$ differences. $C_{m}$ and $C_{a}$ are the angle reward weight and sensor rotation reward weight, respectively. $R_{\mathrm{ext}}$, calculated by the policy-processing layer, is saved in the experience pool together with the training data of the critic network in the targeted decision-making network.

(5) The policy-processing layer feeds $a_{tar}$ and $R_{\mathrm{ext}}$ to the upper-layer network, which stores its action, $a_{reg}$, and external reward $R_{\mathrm{ext}}$ into the experience pool.

(6) The critic network of the two-layer network draws data from the experience pool and then trains and updates the network parameters as in Section 3.1.4.

(7) Each satellite is trained with the network parameters in the cycle of (1)–(6) until convergence.

The training and parameter update process of the MAPPO-RHC algorithm (Algorithm 1) is as follows:
**Algorithm 1:** Training and parameter update process of the MAPPO-RHC algorithm
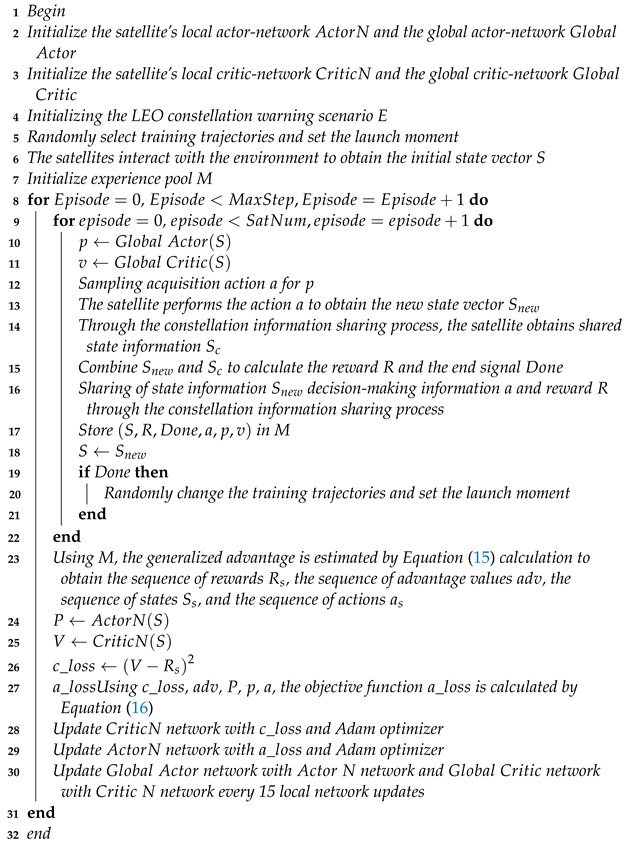


    As shown in Algorithm 1, the algorithm initializes the local actor network Actor, the global actor network Global Actor, the local critic network Critic, and the global critic network Global Critic for each satellite (lines 2 and 3). The LEO constellation’s early warning system is then initialized, resetting the sensor’s pointing direction and the satellite’s position (line 4). *n* training trajectories are randomly selected with different launch points and directions, and the launch moment for each target is set (line 5).

After initialization, the satellites interact with the environment in their initial state and obtain the state information $S=\{T_{num}$, pos, vel, $L_{bit}$, $L_{win}\}$ (line 6); $T_{num}$ is the number of targets. pos is the position of targets. vel is the velocity of targets. and $L_{bit}$ is the bid list of satellites. $L_{win}$ is the list of winners. Iterative training begins, and MaxStep is the maximum number of iteration steps. SatNum is the number of satellites (lines 8 and 9). *S* is preprocessed and input to Global Actor and Global Critic to calculate policy *p* and value *v* (line 10 and 11).

The satellites obtain actions *a* for *p* sampling, interact with the environment to update the state information $S_{new}$, and then obtain the shared information, $S_{c}$, of nearby satellites via inter-satellite communication (lines 12–14). $S_{new}$ and $S_{c}$ are combined to calculate reward *R* and to determine whether tracking should end. Then, $S_{new}$, *a*, and *R* are shared with the other satellites via inter-satellite communication (lines 15, 16).

When satellites have completed one decision-making and information interaction, global policy *P* and value *V* are calculated. Then, the loss coefficient, c_loss, for the critic network and the loss coefficient, a_loss, for the actor network are calculated (lines 23–27). The local networks are updated using the loss coefficients and the Adam optimizer (lines 28, 29). After every 15 updates of the local network parameters, they are replicated to the global network (line 30).

#### 3.2.2. Local Search Algorithm Based on Random Hill Climbing

Reinforcement-learning algorithms are used to improve their decision-making performance by receiving experience from the environment. However, the training set cannot contain all HGV maneuvering trajectories; thus, there is still room for improving the tracking policies generated by reinforcement-learning algorithms for new targets. We design a local search method based on random hill climbing after the hierarchical proximal policy-optimization algorithm, as as shown in Algorithm 2.
**Algorithm 2:** Single-satellite local search method based on random hill climbing**Input**: $P_{SO}$: The initial multi-satellite tracking plan is obtained from a hierarchical   proximal policy-optimization algorithm. ${It}_{lcs}$: The maximum number of   iterations for the local search.**Output**: $P_{SO}$: Final tracking plan
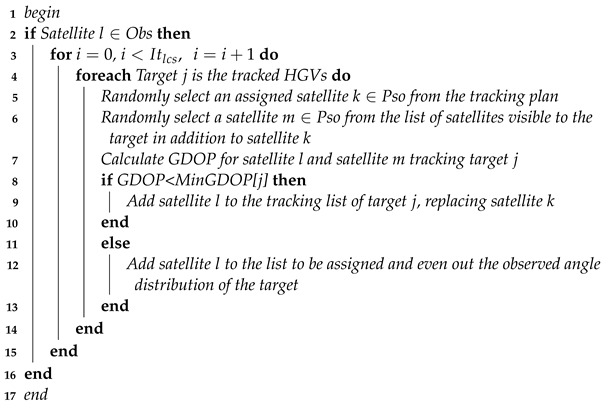


The local search algorithm is run independently in each satellite. In the Algorithm 2, the multi-satellite tracking plan $P_{SO}$ of the hierarchical proximal policy-optimization algorithm and the maximum number of iterations for the local search ${It}_{lcs}$ are used as inputs. The visibility of the satellites to the target is calculated to determine the possibility of tracking the target (line 2). All visible targets are traversed (line 4). Then, an assigned satellite is randomly selected as the proposed replacement satellite from the tracking plan obtained by inter-satellite sharing.

Satellite *m* is randomly selected from the list of satellites visible to the target in addition to satellite *k* (line 6). The GDOP of satellite *l* and satellite *m* to target *j* is calculated. If the GDOP is smaller than the current geometric accuracy factor MinGDOP[j] of this target, satellite *k* in the tracking plan list is replaced with satellite *l*. MinGDOP is the minimum GDOP list. Suppose that the searched satellite does not provide a smaller GDOP. In that case, it is added to the list of other visible targets to be assigned in order to optimize the distribution of the observed angles of the other targets (line 12). The final tracking plan is the output of Algorithm 2.

The computational complexity of the task consensus phase in the constellation information sharing policy is $O\left(\frac{{\left(n^{2}\hat{m}\right)}^{2}}{2}\right)$, $\hat{m}=\max_{i=1}^{n}\;\max_{g=1}^{n}\;\left(m_{ig}\right)$, where *n* is the number of satellites. $m_{ig}$ is the number of communication time windows between two satellites. $\hat{m}$ indicates the maximum number of communication time windows between satellites during the mission. The computational complexity of the independent decision algorithm for each satellite is $O\left(u^{2}+\left(u-1\right)\cdot \left(N_{u}\cdot d_{u}^{2}+N_{l}\cdot d_{l}^{2}\right)+It_{lcs}\cdot u\cdot s^{2}\right)$, where *u* is the number of HGVs; $N_{u}$ and $N_{l}$ are the lengths of the state spaces of the upper neural network and the lower neural network, respectively; $d_{u}$ and $d_{l}$ are the dimensions of each element in the state spaces of the upper and lower neural networks, respectively; and *s* is the number of satellites visible to the HGV. Hence, the computational complexity of the MAPPO-RHC is $O\left(n\cdot \frac{{\left(n^{2}\hat{m}\right)}^{2}}{2}\cdot \left(u^{2}+\left(u-1\right)\cdot \left(N_{u}\cdot d_{u}^{2}+N_{l}\cdot d_{l}^{2}\right)+It_{lcs}\cdot u\cdot s^{2}\right)\right)$.

## 4. Simulation and Analysis

First, the constellation configuration used in this paper and its coverage of different altitudes in near space are described. The parameter settings for the target trajectory in the training set and the target trajectory in the test set are described. Then, the hyperparameter settings of MAPPO-RHC and the training result graphs are provided. Finally, the three algorithms, MAPPO-RHC, MAPPO, and MADDPG, are compared and tested in two different scenarios.

### 4.1. Constellation Parameter Setting

The constellation selection for the LEO constellation warning system must satisfy the global coverage requirements for near space and target at least two-fold coverage requirements and real-time inter-satellite communication requirements. Therefore, the Walker-δ constellation consists of 152 satellites that are evenly distributed in eight orbital planes with a phase difference of four units between adjacent orbital planes.

The satellite orbital altitude $H_{s}$ is 1600 km, and the orbital inclination is $70^{\circ }$. Each satellite carries an IR sensor with a field of view, $\alpha_{field}$, of $3^{\circ }$. The observation angle and the image plane measurement errors of the sensor are equivalent to the measurement angle error, $\sigma_{\theta }$, in the two-dimensional plane of $0.5^{\circ }$. The maximum detection distance, $L_{range}^{\max}$, is obtained according to Equation (3). The maximum speed, $\omega_{search}^{\max}$, of the sensor in the search state is $10^{\circ }$/s, and the maximum speed, $\omega_{sta}^{\max}$, of the imaging state is $5^{\circ }$/s.

The detection distance of the IR sensor is obtained from Equation (3), where $D_{0}=0.3$ m, $D^{*}=1\times 10^{10}$ m· Hz${}^{1/2\;}$· W${}^{-1}$. $\tau_{a}=0.4$, $\tau_{o}=0.35$, $A_{d}=8\times 10^{-10}$ m^2^, $SNR_{\min}=1$.

The limb observation constraint and the maximum detection distance limit the space coverage capability of the sensor. The coverage is a circular area centered on the sub-satellite point. The inner annular radius is the arc distance, $D_{min}$, from the tangent point of the satellite and Earth relative to the sub-satellite point. The outer diameter is the arc distance, $D_{max}$, from the ground projection of the tangent point of the satellite and near outer space relative to the sub-satellite point:

(21) $$D_{\min}=\left[\cos^{-1}\left(\frac{R_{e}}{R_{e}+H_{s}}\right)-\cos^{-1}\left(\frac{R_{e}}{R_{e}+H_{tar}}\right)\right]\cdot R_{e}$$

(22) $$D_{\max}=\cos^{-1}\left[\frac{{\left(R_{e}+H_{tar}\right)}^{2}+{\left(R_{e}+H_{s}\right)}^{2}-L{{}_{range}^{\max}}^{2}}{2\cdot \left(R_{e}+H_{tar}\right)\cdot \left(R_{e}+H_{s}\right)}\right]\cdot R_{e}$$

where $H_{tar}$ has the range [20∼100 km], and $R_{e}$ is the radius of Earth.

**Figure 8:**
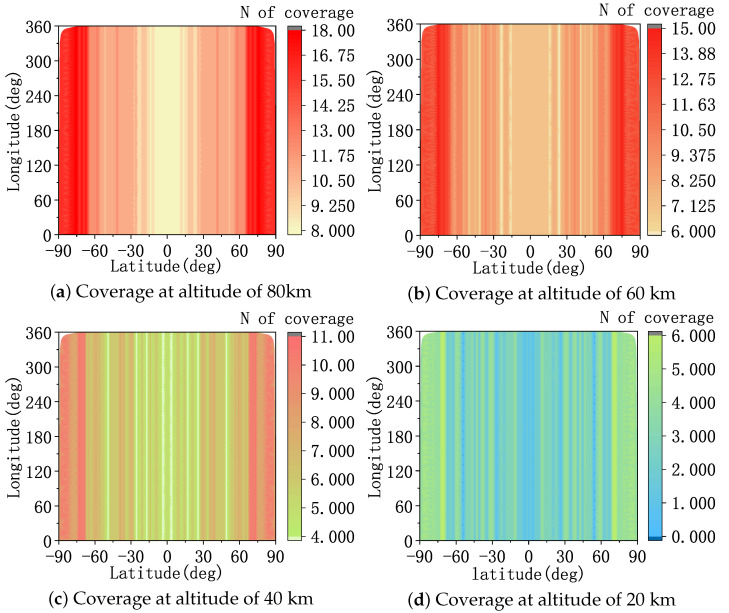
The coverage of near space by the constellation.

As shown in Figure 8, the constellation has good coverage of targets at different altitudes in the near space area. The coverage of the Walker-δ constellation is latitude-sensitive and becomes larger as the latitude increases. For HGVs above 40 km, the constellation can achieve at least four-fold coverage. An altitude of 20 km serves as the near-Earth edge of near space, and the sensor is constrained by limb observations with a small viewing angle. The constellation can still provide more than two-fold coverage at middle and high latitudes for the near-earth limit altitude. It can be seen that the constellation selected in this paper meets the coverage requirements for the targets in near space.

### 4.2. Generating the Training Set

The training set includes the trajectories of HGVs distributed in different positions worldwide. The initial parameters of the trajectories include latitude and longitude, altitude, direction, and velocity. Among them, the initial latitude and longitude of the target are randomly generated in the global range, the initial altitude is randomly generated in the range of 80∼60 km, the initial direction is randomly selected in 0∼$360^{\circ }$, and the initial velocity is randomly selected in 6000∼7000 m/s. During training, 2∼4 trajectories are randomly selected from the training set each time to form a training scenario.

The network and training hyper-parameters are set as shown in Appendix A.

The neural network was trained on a computer equipped with an i7-8700 processor, 16 g of RAM, and an NVIDIA GTX 1060 graphics card. The PyTorch deep-learning framework and the PyCharm simulation platform were used, and the simulation language is Python.

### 4.3. Training Results

**Figure 9:**
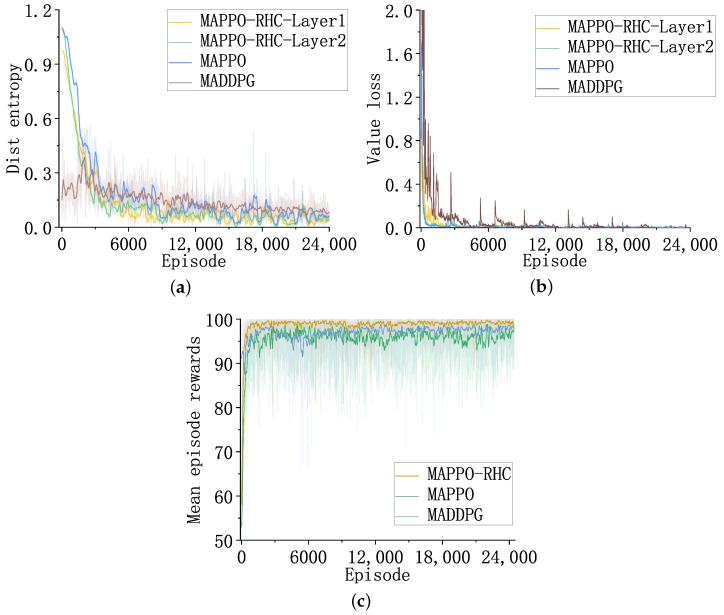
The results of training. (**a**) The distributional entropy of the actor network. (**b**) The loss of value of the critic network. (**c**) The average reward.

Figure 9 shows the curves of the distribution entropy of the actor network, the loss of the critic network value, and the average gain of the three algorithms, MAPPO-RHC, MAPPO, and MADDPG, during the training process.

The MAPPO-RHC-layer1 curve in Figure 9a indicates the degree of clarity of the region’s decision-making network in selecting search regions. The lower the distribution entropy, the more concentrated the probability distribution of the actions that are outputted from the network. Among them, the distribution entropy of the MAPPO-RHC algorithm converges the quickest. The PPO algorithm converges slower than MAPPO-RHC in the first 800 episodes, and the final convergence accuracies are similar.

However, during the training process, the DDPG algorithm converges slower than the other two algorithms with respect to distribution entropy. The convergence value is larger than the other two algorithms. The MAPPO-RHC algorithm has advantages in terms of clarity during target selection. As shown in Figure 9b, the value loss curve of the critic network indicates the accuracy of the evaluation of the actor network’s output results.

The smaller the value loss, the more the critic network evaluates the actor network’s output accurately. The value loss curves of MAPPO-RHC-layer2 and MAPPO have the best convergence. The evaluation function of MAPPO-RHC-layer1 is shown in Equation (20). The discrete evaluation causes the evaluation function to be unsmooth; therefore, the oscillation is more evident at the beginning of training, and the convergence trend after 2000 episodes is close to that of MAPPO-RHC-layer2.

The convergence speed of the MADDPG algorithm is slow. The spikes in the value loss curve are caused by a sudden change in the relative states of satellites and targets after the old targets disappear or the new targets appear. The spikes diminish rapidly after a few episodes, and the effect of old and new targets’ change on the network gradually disappears as training times increase.

In Figure 9c, the average reward of the algorithm represents the average of the external reward obtained by the satellite during the tracking task. The MAPPO-RHC algorithm converges the quickest, and the average reward is always higher than the other two algorithms, eventually converging to 99. The MAPPO and MADDPG algorithms converge similarly, and the average reward increases alternately, eventually converging to 97.

Next, we simulate and analyze the MAPPO-RHC algorithm in two different scenarios with four intelligent satellite decision algorithms that have been researched in recent years. The MADDPG multi-satellite mission-scheduling algorithm is an improved intelligent satellite decision-making algorithm proposed by Huang et al. 2021, which solves the coherent scheduling problem with multiple constraints [28].

The multi-satellite, multi-target coherent tracking decision-making problem researched in this paper is also a coherent scheduling problem with multiple constraints; therefore, the MADDPG algorithm is used as the comparison algorithm of MAPPO-RHC. The MAPPO multi-satellite online scheduling algorithm is a satellite intelligent decision-making algorithm proposed by Li et al. in 2022, which effectively solves the satellite resource conflict and time-window-conflict problem [26]. The MAPPO algorithm is used as the comparison algorithm considering that the MAPPO-RHC algorithm is an improved algorithm based on MAPPO, and the performance difference of the improved part of the algorithm can be compared more intuitively.

The HT3O algorithm is a hierarchical reinforcement learning network based on the MADDPG and deep Q-network (DQN). HT3O is used as a comparison algorithm, where MADDPG is used for the regional decision-making network, and DQN is used for the targeted decision-making network [30]. The hybrid genetic parallel tabu (HGPT) algorithm is based on a multi-layer interactive task-planning framework that combines the genetic annealing algorithm, parallel tabu algorithm, and heuristic rules to achieve collaborative multi-resource task allocation, planning, and scheduling [8].

In the first scenario, the sequential launch of three groups of HGVs with three targets each and a dense distribution of targeted flight paths occurs. In the second scenario, three groups of HGVs are sequentially launched, with four targets per group. The trajectories of each group of targets are scattered and distributed. The specific test results are described below.

### 4.4. Test Scenario 1

The test set consists of nine trajectories of HGVs, which were sequentially launched in groups of three. The parameters of HGVs are shown in Appendix B.1.

**Figure 10:**
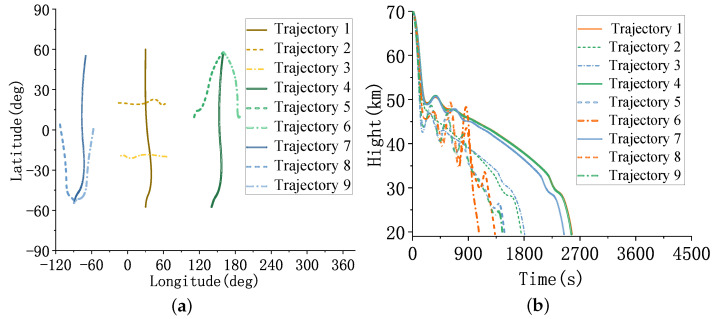
The trajectories of HGVs. (**a**) The latitude and longitude of the trajectories. (**b**) The altitude of the trajectories.

As shown in Figure 10: trajectory 1, trajectory 2, and trajectory 3 cross in the middle of the flight. Trajectory 4, trajectory 5, and trajectory 6 move from north to south to the same target’s position. Trajectory 7, trajectory 8, and trajectory 9 start from the same initial position and move from north to south directions relative to different targeted positions.

**Figure 11:**
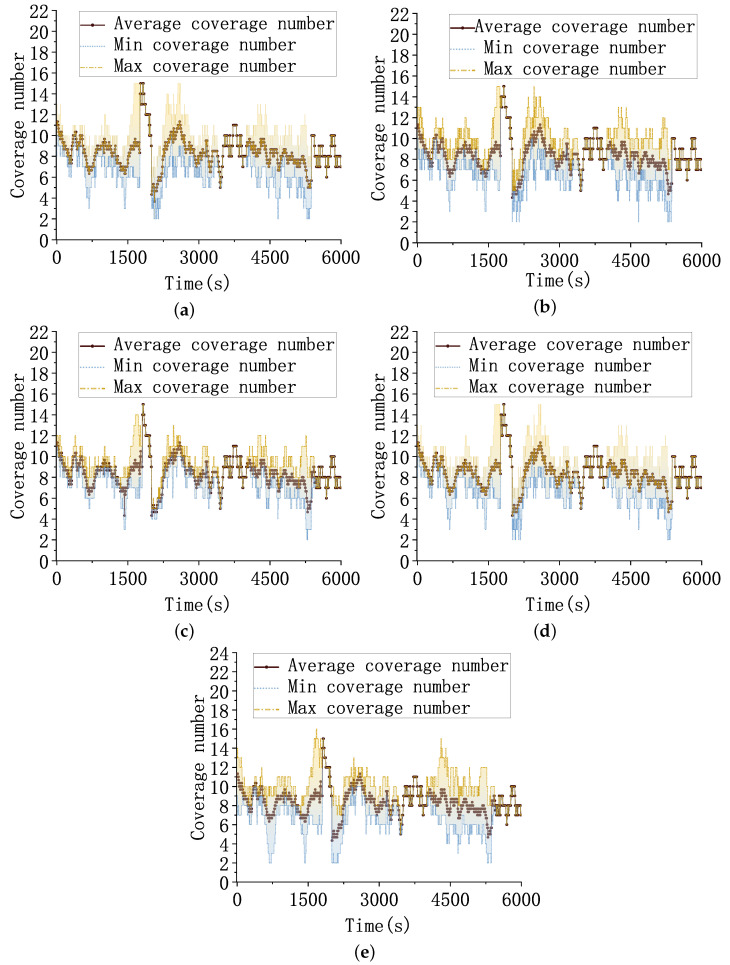
Target covered folds. (**a**) Coverage folds of the MADDPG algorithm for the targets. (**b**) Coverage folds of the MAPPO algorithm for the targets. (**c**) Coverage folds of the MAPPO-RHC algorithm for the targets. (**d**) Coverage folds of the HT3O algorithm for the targets. (**e**) Coverage folds of the HGPT algorithm for the targets.

As shown in Figure 11, the horizontal coordinate is the simulation time and the vertical coordinate is the coverage folds of the constellation on the target. The yellow line indicates the maximum coverage folds, the brown line indicates the average coverage folds, and the blue line indicates the minimum coverage folds. To achieve dual-satellite positioning, each target needs to be covered by at least two satellites at the same time. As seen in the figure, all five algorithms can meet the minimum number of observation satellites required for the dual-satellite positioning algorithm.

Since the visibility of the constellation to the targets at the same time is fixed and computable, the smaller the difference between the maximum coverage fold and the minimum coverage fold, the more balanced the coverage of the constellation to the targets. The distance between the targets and the flight altitude varies continuously with time. When the target is at a higher flight altitude, the target visibility from the satellite is better, and so the coverage fold curve appears as a spike. When the targets are closer or at lower flight altitudes, the visibility of the constellation to the targets is weak, and the coverage fold curve shows a trough. At this time, the coverage balance of the constellation to the target is different due to the different searching abilities of intelligent decision algorithms.

The cross-trajectory 1∼3 fly to similar airspaces at 2000∼2100 s, which are relatively close to each other. The available resources in this airspace need to be scheduled to three targets; thus, the coverage fold of each target is low, and the resultant curves of all three algorithms show a trough. The HGPT algorithm has no preparatory work, such as pre-training and the future maneuvering capability of the target is unknown in the real-time scenario, resulting in a large uncertainty of the future trajectory of the target. The HGPT algorithm has the worst resource allocation balance because of the large limitation of its merit-seeking ability when making decisions based on real-time data.

The flight times of the targets are different, and only one target in flight exists in the scenario when the three curves overlap. At around 5000 s, trajectories 1–3 gradually decrease the available observation resources in the constellation as the flight altitude decreases. The minimum coverage folds of HGPT, MADDPG, MAPPO, and HT3O algorithms show multiple and longer periods of two to three coverage folds. The minimum coverage folds of the MAPPO-RHC algorithm can achieve at least four folds most of the time, although it also shows two folds for 5 s, which shows that the algorithm has a more uniform resource allocation.

The coverage fold curves show that the satellites can achieve complete multi-fold tracking observations for each target. The geometric positioning accuracy of the dual-satellite algorithm depends on the observation angle. The MAPPO-RHC algorithm optimizes the coverage angle distribution to improve the geometric positioning accuracy, especially when fewer resources are available. The five algorithms’ average GDOP and positioning error profiles are shown below.

**Figure 12:**
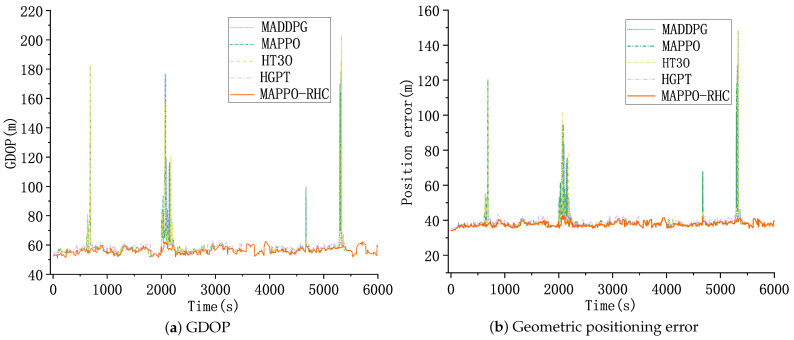
GDOP and geometric positioning accuracy.

As shown in Figure 12, Figure 12a shows the curve of GDOP with time. The horizontal coordinate is the simulation time and the vertical coordinate is the GDOP. The GDOP of all five algorithms grows around 800, 2100, 4600, and 5400 s as the constellation coverage of the target becomes weaker due to the crossed trajectory or low altitude of the target. Benefiting from the ability of hierarchical reinforcement learning to maximize the global reward and the improved solution-space search capability of the local search algorithm, the MAPPO-RHC algorithm has an advantage when the constellation coverage is weak, and the increase in GDOP of the MAPPO-RHC algorithm is flatter, all within 70.

Figure 12b shows the geometric positioning error of the targets. The trend of the geometric positioning error of the five algorithms is the same as that of the GDOP curve. The geometric positioning errors of the MAPPO-RHC algorithm are within 50 m and between 35 and 40 m at all moments except for the four spikes.

The calculation time of the intelligent algorithm is a visual representation of the real-time performance of the algorithm. The average calculation time of the single-satellite neural network in the decision-making process, the average time of single-satellite decision making, and the average time of local search in the MAPPO-RHC algorithm were counted. As shown in Table 2, $D_{\mathrm{Network}}^{\mathrm{MAPPO}–\mathrm{RHC}}$, $D_{\mathrm{Network}}^{\mathrm{MAPPO}}$, $D_{\mathrm{Network}}^{\mathrm{MADDPG}}$, and $D_{\mathrm{Network}}^{\mathrm{HT}3O}$ are the neural networks of the four algorithms for the calculation delay. $D_{\mathrm{Decision}}^{\mathrm{MAPPO}–\mathrm{RHC}}$, $D_{\mathrm{Decision}}^{\mathrm{HT}3O}$, $D_{\mathrm{Decision}}^{\mathrm{MAPPO}}$, $D_{\mathrm{Decision}}^{\mathrm{HGPT}}$, and $D_{\mathrm{Decision}}^{\mathrm{MADDPG}}$ are the single-satellite single decision calculation delay for each of the three algorithms. $D_{\mathrm{Search}}^{\mathrm{MAPPO}–\mathrm{RCH}}$ is the local search calculation delay of the MAPPO-RHC algorithm.

As shown in Table 2, the neural network’s calculation is the core calculated part of the algorithm in the single-satellite-tracking decision process, and its time calculation directly affects the real-time results of the entire decision-making process. MAPPO-RHC takes PPO as the base algorithm and expands the network structure into a regional decision-making network and a target decision-making network, which has a more complex network structure. MAPPO-RHC gives the computation time of the neural network certain advantages over MAPPO algorithm by reducing the solution space. MAPPO-RHC needs to preprocess the input data of the lower network according to the action of the output of the upper network.

Therefore, the computation time for a single decision is higher than that of intelligent algorithms, such as MAPPO and MADDPG that do not require hierarchical decision making. As seen in Table 2, although MADDPG takes less time to compute, its positioning accuracy is slightly lower than MAPPO-RHC as seen in the previous simulation results. HT3O has a slightly larger computation time than MADDPG because the network structure is more complex than MADDPG.

The iterative screening process of HGPT leads to a higher computation time when compared with other intelligent decision algorithms. The calculation time of the local search algorithm of MAPPO-RHC is shorter than the calculation time of the neural network in the four intelligent algorithms. Moreover, adding the heuristic algorithm avoids invalid and repeated searches, which further improves the calculation efficiency of the local search algorithm.

### 4.5. Test Scenario 2

The test set consists of 12 trajectories of HGVs, which were sequentially launched in groups of three. The parameters of HGVs are shown in Appendix B.2.

**Figure 13:**
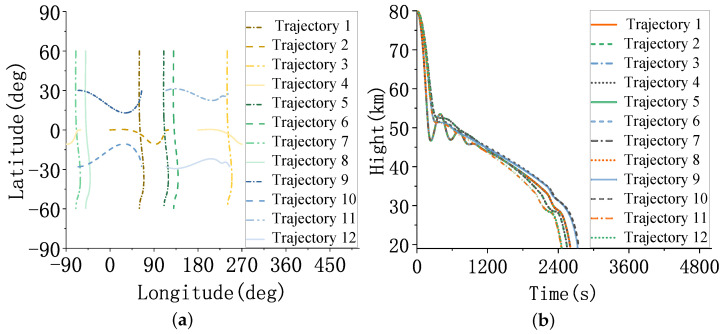
The trajectories of HGVs. (**a**) The latitude and longitude of the trajectories. (**b**) The altitude of the trajectories.

The trajectories’ latitude, longitude, and altitude are shown in Figure 13.

As shown in Figure 13, trajectories 1∼4 are two groups of cross trajectories, and trajectories 5∼8 are two groups of parallel trajectories distributed in two different geographical positions that are $180^{\circ }$ apart in longitude. Trajectories 9∼12 have a latitude distribution of $\pm 40^{\circ }$.

**Figure 14:**
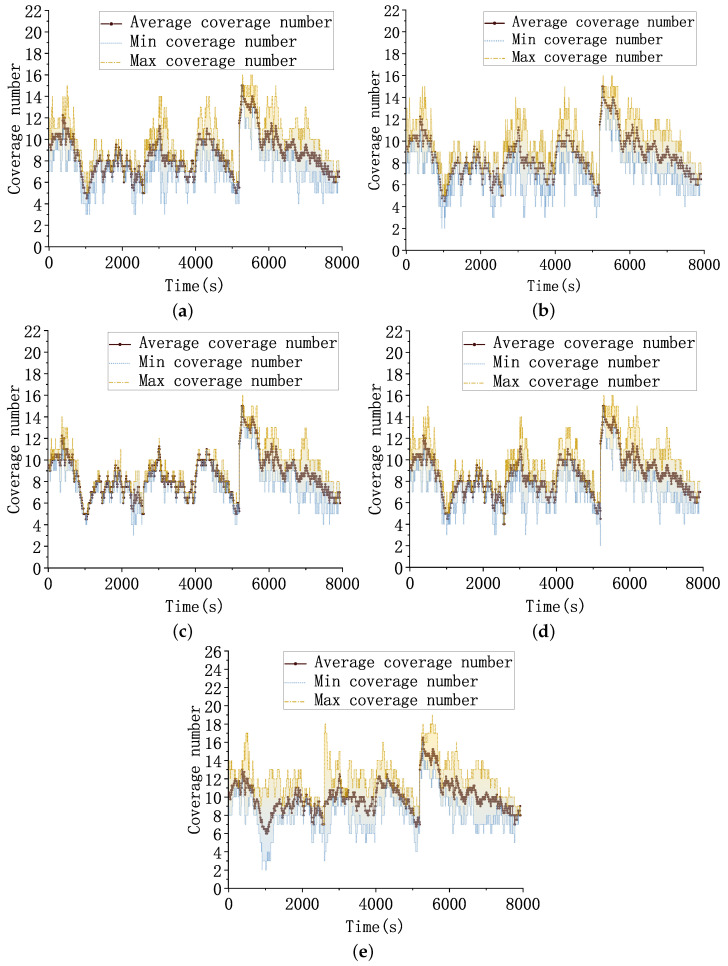
Target covered folds. (**a**) Coverage folds of the MADDPG algorithm for the targets. (**b**) Coverage folds of the MAPPO algorithm for the targets. (**c**) Coverage folds of the MAPPO-RHC algorithm for the targets. (**d**) Coverage folds of the HT3O algorithm for the targets. (**e**) Coverage folds of the HGPT algorithm for the targets.

As shown in Figure 14, all five algorithms can satisfy more than two-fold coverage for the target. Test scenario 2 has two sets of HGVs trajectories, two sets of trajectories exist in the scenario at the same time, and the two sets of trajectories are distributed in geographically distant locations. Therefore, test scenario 2 requires the scheduling of more satellites in the constellation to track all targets in the scenario simultaneously. Similar to test scenario 1, each set of targets still has spatial relationships, such as crossing or parallel. Therefore, the visibility of the constellation to the targets is weak when the targets are close together or at low altitudes.

At the 1050 s moment, four targets cross two by two, the target positions are concentrated, and fewer resources can be scheduled. Therefore, the coverage fold curve has a low valley. At that time, the minimum coverage fold of the MAPPO and HGPT algorithms is 2. The MADDPG and HT3O can achieve three- to four-fold coverage. The MAPPO-RHC algorithm can almost retain at least four satellites by observing a target simultaneously during the simulation, and the observation satellites are more evenly distributed. Throughout the simulation, the minimum coverage fold of MAPPO-RHC algorithm is generally higher than that of the baseline algorithm.

**Figure 15:**
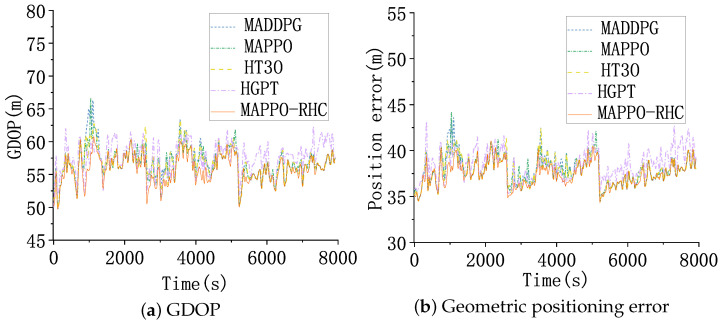
GDOP and geometric positioning accuracy.

Moreover, the difference between the maximum coverage fold and the minimum coverage fold is smaller, which shows a more balanced resource allocation. As shown in Figure 15, Figure 15a shows the curve of GDOP with time. The GDOP of all five algorithms grows at around 800, 2100, 4600, and 5400 s. Compared to test scenario 1, the distribution of targets in test scenario 2 is more dispersed. Although the distributions of the target trajectories were more dispersed, the GDOP and geometric positioning accuracy of the five algorithms did not fluctuate significantly, benefiting from the fact that the reward function of the intelligent decision algorithm considers the loss caused by the large angle rotation of the sensor and the mechanism to encourage early back-scan to the vicinity of the target.

The GDOP of the MAPPO-RHC algorithm still has an advantage, within 70. Figure 15b shows the geometric positioning error of the targets. The trend of the geometric positioning error of the five algorithms is the same as that of the GDOP curve. The geometric positioning errors of the MAPPO-RHC algorithm are within 50 m and remain between 35 and 40 m at all times, with the exception of four spikes.

Then, as in Scenario 1, the average calculation time of the single-satellite neural network during decision making, the average time for single-satellite decision making, and the average time of the local search in the MAPPO-RHC algorithm were counted.

As shown in Table 3, the complexity of the number of targets and position distribution in scenario 2 is higher than that in scenario 1. The input data of the neural network of the satellite includes a variety of targets, such as observable and those worthy of early back-scan; therefore, the computational delay of the neural network is generally slightly higher. However, the number of targets in the local area is less than that of the test scenario 1, the region decision-making network can significantly reduce the solution space, and the data processing latency of the decision-making processing layer becomes lower.

Therefore, the single-satellite single decision-making delay of both MAPPO-RHC and HT3O algorithms is slightly lower than that of scenario 1. The average calculation time of the single-satellite decision-making of the MAPPO-RHC algorithm is similar to that of MAPPO, which is 0.013 s. The calculation time of the decision-making algorithm after adding a local search is still not inferior to that of the MAPPO algorithm.

## 5. Conclusions

In this paper, the cooperative positioning of a LEO constellation on HGVs was used as the research background. A geometric positioning model of multiple targets in near space was developed for the constellation warning system. A hierarchical proximal policy-optimization algorithm was proposed, which effectively reduces the solution space of the algorithm. Finally, a random hill-climbing search algorithm was added after the hierarchical proximal policy-optimization algorithm to improve the solution-space search capability of the tracking and decision-making algorithm.

The minimum coverage folds of MAPPO-RHC were generally better than that of the baseline algorithm. This is beneficial for the optimization capability of the hierarchical proximal policy-optimization algorithm with respect to the distribution entropy of the observation angle and the local search capability of the random hill-climbing algorithm. The MAPPO-RHC algorithm improved geometric positioning accuracies. As a result, the MAPPO-RHC algorithm provides a feasible solution for the real-time decision-making problem of the LEO early warning constellation.

However, the method used in this paper also has the following limitations:

(1) The positioning error can be further reduced by the filtering algorithm after geometric positioning. Since different filtering algorithms introduce different improvements with respect to the positioning accuracy for different scenarios, in this paper, GDOP and geometric positioning errors were used as targets for optimization and comparison to reflect the decision-making algorithm’s performance more intuitively.

(2) Restricted by the fixed input dimension of the neural network, the number of targets in the input matrix of the targeted decision-making network was fixed. When the number of targets satisfying the observation constraint was larger than the fixed value, this paper determined alternative tracking targets by screening the rewards. In the dynamic coherent tracking problem with multiple satellites and multiple targets, there is room for improving the screening capability of the evaluation function.

For future research topics, there are two extended research directions worth exploring. (1) Considering the influence of the filtering algorithm on positioning accuracy under different observation conditions, designing a decision-making network that can consider the filtering accuracy is a worthy direction for future research. (2) When the number of targets satisfying the satellite observation constraints is more than the number of alternative targets in the target decision-making network, the filtering capability of the evaluation function is weak. Therefore, designing a network that can adapt to dynamically adjusting the number of alternative tracking targets or a target-screening method is worth exploring. 

## Figures and Tables

**Table 1:** Observation angle score.

	$\alpha_{\mathrm{az}}^{\mathrm{inc}}$	$\left[-30^{\circ },30^{\circ }\right)$	$\left[30^{\circ },150^{\circ }\right)$	$\left[150^{\circ },270^{\circ }\right)$	$\left[-150^{\circ },-30^{\circ }\right)$
$I_{\mathrm{vis}}$					
True		40	60	40	60
False		10	20	20	0

**Table 2:** Algorithmic calculation delay.

Statistics (s)	$D_{\mathrm{Network}}^{\mathrm{MAPPO}–\mathrm{RHC}}$	$D_{\mathrm{Network}}^{\mathrm{MAPPO}}$	$D_{\mathrm{Network}}^{\mathrm{MADDPG}}$	$D_{\mathrm{Network}}^{\mathrm{HT}3O}$	$D_{\mathrm{Decision}}^{\mathrm{MAPPO}–\mathrm{RHC}}$
Maximum	0.0041	0.0068	0.0031	0.0030	0.019
Average	0.0028	0.0038	0.0012	0.0010	0.017
Minimum	0.0019	0.0028	0.0005	0.005	0.015
	$D_{\mathrm{Decision}}^{\mathrm{HT}3O}$	$D_{\mathrm{Decision}}^{\mathrm{MAPPO}}$	$D_{\mathrm{Decision}}^{\mathrm{MADDPG}}$	$D_{\mathrm{Decision}}^{\mathrm{HGPT}}$	$D_{\mathrm{Search}}^{\mathrm{MAPPO}–\mathrm{RCH}}$
Maximum	0.017	0.0091	0.0068	0.7564	0.0014
Average	0.015	0.0073	0.0048	0.2639	0.0002
Minimum	0.014	0.0047	0.0027	0.2508	0

**Table 3:** Algorithmic calculation delay.

Statistics (s)	$D_{\mathrm{Network}}^{\mathrm{MAPPO}–\mathrm{RHC}}$	$D_{\mathrm{Network}}^{\mathrm{MAPPO}}$	$D_{\mathrm{Network}}^{\mathrm{MADDPG}}$	$D_{\mathrm{Network}}^{\mathrm{HT}3O}$	$D_{\mathrm{Decision}}^{\mathrm{MAPPO}–\mathrm{RHC}}$
Maximum	0.0063	0.0077	0.0042	0.0048	0.022
Average	0.0041	0.0043	0.0015	0.0020	0.013
Minimum	0.0029	0.0029	0.0006	0.0010	0.007
	$D_{\mathrm{Decision}}^{\mathrm{HT}3O}$	$D_{\mathrm{Decision}}^{\mathrm{MAPPO}}$	$D_{\mathrm{Decision}}^{\mathrm{MADDPG}}$	$D_{\mathrm{Decision}}^{\mathrm{HGPT}}$	$D_{\mathrm{Search}}^{\mathrm{MAPPO}–\mathrm{RCH}}$
Maximum	0.019	0.022	0.015	0.2274	0.0048
Average	0.011	0.013	0.007	0.1633	0.0005
Minimum	0.006	0.008	0.003	0.1581	0

## Data Availability

Not applicable.

## Appendix A. Hyper-Parameter Settings for Networks and Training

The network parameters and hyper-parameters of training are set as shown in Table A1.

**Table A1:** MAPPO-RHC/PPO parameter setting.

Episode Length	Hidden Size	Layer N	Gain
152	64	3	0.1
**lr**	**ppo Epoch**	**Clip Param**	**Gamma**
$5\times 10^{-5}$	15	0.2	0.99

In Table A1, ‘episode length’ is the single training length, ‘hidden size’ is the hidden layer dimension of the network, ‘layer N’ is the number of layers of the network, ‘gain’ is the gain at the output of the actor-layer, ‘lr’ is the learning rate of the actor network and critic network, ‘ppo epoch’ is the number of times each Episode is learned, ‘clip param’ is the threshold factor for the importance weight, and ‘gamma’ is the reward discount rate.

In Table A2, ‘learning start step’ is the number of steps needed to accumulate experience before training, ‘memory size’ is the amount of data in the experience pool, and ‘max grad norm’ is the maximum gradient norm.

**Table A2:** DDPG parameter setting.

Episode Length	Hidden Size	Layer N	Learning Start Step
300	64	3	100
**lr**	**Memory Size**	**Max Grad Norm**	**Gamma**
$1\times 10^{-2}$	$1\times 10^{6}$	0.5	0.97

## Appendix B. The Parameters of HGVs

### Appendix B.1. Test Scenario 1

As shown in Table A3, every two groups of trajectories are sequentially connected, trajectory 1, trajectory 2, and trajectory 3 are cross trajectories, and the initial direction of trajectory 1 is perpendicular to trajectory 2 and trajectory 3. Trajectory 4, trajectory 5, and trajectory 6 are similar target point trajectories flying from different initial positions to similar target positions. Trajectory 7, Trajectory 8, and Trajectory 9 have the same starting position and move in different directions from the same initial positions. The trajectories’ latitude, longitude, and altitude are shown in the figure below.

**Table A3:** HGV parameter settings.

$T_{\mathrm{begin}}\left(s\right)$	$\mathrm{Lo}n_{\mathrm{in}}\left({}^{\circ }\right)$	$\mathrm{La}t_{\mathrm{in}}\left({}^{\circ }\right)$	$H_{\mathrm{in}}\left(m\right)$	$\mathrm{Ve}l_{\mathrm{in}}\left(m/s\right)$	$\mathrm{Hea}d_{\mathrm{in}}\left({}^{\circ }\right)$	$\mathrm{Lo}n_{\mathrm{tar}}\left({}^{\circ }\right)$	$\mathrm{La}t_{\mathrm{tar}}\left({}^{\circ }\right)$	$H_{\mathrm{tar}}\left(\mathrm{km}\right)$	$\mathrm{Ve}l_{\mathrm{tar}}\left(m/s\right)$
1	30	60	70,000	7000	180	30.1	−57.6	19,479	2000.4
1	−15	20	70,000	6500	89	64.5	19.9	19,189	2000.4
1	65	−20	70,000	6500	−89	−14.5	−19.9	19,418	2000.5
1808	160	58	70,000	7000	190	140.3	−57.6	19,445	2000.1
1808	160	58	70,000	6500	225	111.2	9.4	19,390	2000
3295	160	58	70,000	6500	135	187.6	9.7	20,760	2000.5
3295	−70	55	70,000	7000	190	−89.7	−54.6	19,507	2000.7
3295	−113	4	70,000	6500	160	−89.8	−54.6	19,521	2000.1
4320	−57	0.7	70,000	6500	200	−89.4	−54.7	19,447	1999.8

### Appendix B.2. Test Scenario 2

**Table A4:** HGV parameter settings.

$T_{\mathrm{begin}}\left(s\right)$	$\mathrm{Lo}n_{\mathrm{in}}\left({}^{\circ }\right)$	$\mathrm{La}t_{\mathrm{in}}\left({}^{\circ }\right)$	$H_{\mathrm{in}}\left(m\right)$	$\mathrm{Ve}l_{\mathrm{in}}\left(m/s\right)$	$\mathrm{Hea}d_{\mathrm{in}}\left({}^{\circ }\right)$	$\mathrm{Lo}n_{\mathrm{tar}}\left({}^{\circ }\right)$	$\mathrm{La}t_{\mathrm{tar}}\left({}^{\circ }\right)$	$H_{\mathrm{tar}}\left(\mathrm{km}\right)$	$\mathrm{Ve}l_{\mathrm{tar}}\left(m/s\right)$
1	60	60	80,000	7000	180	60.1	−59.6	19,472	2000.4
1	0	0	80,000	7000	89	119.6	0	19,348	1999.9
1	240	60	80,000	7000	180	240	−59.6	19,472	2000.4
1	180	0	80,000	7000	89	−60.4	0	19,348	1999.9
2549	110	60	80,000	7000	180	110.1	−59.6	19,472	2000.4
2549	130	60	80,000	7000	180	130.1	−59.6	19,472	2000.4
2549	290	60	80,000	7000	180	−69.9	−59.6	19,472	2000.4
2549	310	60	80,000	7000	180	−49.9	−59.6	19,472	2000.4
5150	−65	30	80,000	7000	89	64.8	29.6	19,230	2000
5150	−65	−28	80,000	7000	89	64.8	−27.6	19,313	2000.3
5150	115	38	80,000	7000	80	244.6	29.7	19262	2000.8
5150	115	−28	80,000	7000	100	244.6	−27.7	19,240	2000.7
